# Graphical Depiction of Statistical Information Improves Gambling-Related Judgments

**DOI:** 10.1007/s10899-019-09860-1

**Published:** 2019-05-27

**Authors:** Alexander C. Walker, Madison Stange, Mike J. Dixon, Derek J. Koehler, Jonathan A. Fugelsang

**Affiliations:** Department of Psychology, University of Waterloo, Waterloo, ON N2L 3G1 Canada

**Keywords:** Gambling, Debiasing, Unclaimed prize information, Scratch cards, Visual aids

## Abstract

The domain of gambling is rife with both diagnostic and non-diagnostic information. Previous studies examining scratch card gambling have demonstrated that people are often biased by intuitively appealing, yet non-diagnostic information (i.e., unclaimed prize information). The current study investigated how varying the presentation format of a diagnostic piece of information (i.e., payback percentage) could influence participants’ use of this information when in conflict with unclaimed prize information. We hypothesized that when payback percentage information was presented in a graphical, as opposed to a numerical format, participants would be better at ignoring unclaimed prize information and correspondingly have their preferences become congruent with the true value of the presented scratch cards. In Experiment 1 (*N* = 201), with payback percentage presented in a numerical format, participants displayed a non-optimal preference for cards with greater numbers of unclaimed prizes and lower payback percentages. This preference was reversed in Experiment 2 (*N* = 201) when payback percentage was presented in a graphical format. In conclusion, the results of the current study demonstrate how judgments in a scratch card gambling domain can be improved by simply changing the presentation format of a single piece of information.

**Electronic supplementary material:**

The online version of this article (10.1007/s10899-019-09860-1) contains supplementary material, which is available to authorized users.

## Introduction

The ability to decipher informative from uninformative information is an important skill to have in today’s information age. That is, a good decision maker must not only be able to utilize relevant information, but also be able to avoid being misled by the plethora of irrelevant information at their fingertips. Research on human decision making has identified peoples’ frequent shortcomings in this area, exhibiting how clearly irrelevant and non-diagnostic information can influence our decisions (Ariely et al. 2003; Meyvis and Janiszewski 2002; Tversky and Kahneman 1974, 1981; Van Osselaer et al. 2004). For example, people have been shown to make decisions under uncertainty by first anchoring to an irrelevant cue and then adjusting (often insufficiently) away from these anchors, resulting in biased responses caused by this irrelevant information (Ariely et al. 2003; Tversky and Kahneman 1974). Not only does irrelevant information bias decision making when this information is readily available, but people will go so far as to *seek out* non-diagnostic information, even enduring costs in order to obtain it (Baron et al. 1988).

The undue biasing effects of non-diagnostic information can also be observed in the heavily incentivised domain of gambling. For example, upon experiencing repeated losing outcomes in a game of chance, an individual endorsing the gambler’s fallacy will believe that a win is more likely to occur even though in reality each outcome is probabilistically independent of the next (Clotfelter and Cook 1993; Rogers 1998). Since the gambler’s fallacy involves the misattribution of diagnostic value to previous outcomes that bear no influence on future outcomes, it serves as an example of individuals being unduly biased by non-diagnostic information. Furthermore, within the realm of lottery gambling, it is common for players to report using specific strategies and number combinations when choosing their numbers (Holtgraves and Skeel 1992; Turner 2010) despite the fact that none of these tactics will change the odds of winning a traditional draw-based lottery game. Therefore, in the domain of gambling, salient yet non-diagnostic information is often viewed as valuable and can unjustifiably influence an individual’s gambling behaviour. Nevertheless, the gambling domain also features relevant (albeit often complex) information that can assist gamblers in making judgments. One may wonder whether making this information easier to utilize may help gamblers be less influenced by non-diagnostic information, therefore optimizing their gambling-related judgments.

### Unclaimed Prize Information Bias

Within a scratch card game, unclaimed prize information informs gamblers of the number of prizes still available to be won at each prize level. This information is made available online through all American, Canadian, and United Kingdom lottery operators, with most operators updating this information weekly or daily. Despite its intuitive appeal, unclaimed prize information offers no advantage to the gambler attempting to maximize their chance at monetary gain. To understand its lack of utility, imagine making a choice between two scratch card games, Scratch Card A and Scratch Card B. Unclaimed prize information informs you that Scratch Card A has ten $1000 prizes remaining, whereas for Scratch Card B, only a single $1000 prize remains. While this information may *appear* to be diagnostic, it does not allow you to conclude that Scratch Card A is of a higher expected value compared to Scratch Card B. Imagine for example that Scratch Card A (with ten $1000 prizes remaining) has 10,000 cards available for purchase, whereas Scratch Card B (with a single $1000 prize remaining) has only 10 cards available for purchase. Therefore, despite discrepancies in the number of unclaimed prizes available, it remains unknown to the player which scratch card offers the best chance at winning a $1000 prize. In conclusion, while unclaimed prize information may allow a gambler to avoid purchasing a scratch card in which a preferred prize is no longer available (i.e., it may inform players that a specific top prize has already been won), it is useless to the gambler looking to purchase the scratch card affording the greatest chance of monetary gain.

Despite its lack of utility, unclaimed prize information has been shown to bias people’s scratch card related judgments (Walker et al. 2018). Specifically, when presented with three otherwise identical scratch card games, participants felt more likely to win, were more excited to play, and preferred to hypothetically purchase the scratch card game with the greatest number of unclaimed prizes. Additionally, a majority of participants reported that they would be willing to pay to obtain unclaimed prize information, demonstrating that they valued this information despite its lack of utility (Stange et al. 2018).

Unclaimed prize information bias has been shown to persist even when participants are explicitly informed of the total number of scratch cards remaining (Walker et al. 2018). When used together, information regarding the number of unclaimed prizes and the total number of cards remaining allow participants to calculate payback percentage, a diagnostic piece of information indicating the amount of money bet on a certain game that is, on average, paid out as prizing. When participants are presented with scratch cards that possess identical payback percentages, yet vary in the number of unclaimed prizes, they show a preference for scratch cards with more unclaimed prizes. Even worse, given the choice between scratch cards with lower payback percentages but more unclaimed prizes, and cards with higher payback percentages but fewer unclaimed prizes, participants still prefer the scratch cards with more unclaimed prizes. Normatively speaking, participants should show no preference between scratch cards when payback percentages are equivalent, and a preference for scratch cards with fewer unclaimed prizes when this corresponds to a higher payback percentage. Therefore, the presentation of unclaimed prize information seemingly presents a real-world example where individuals are biased by non-diagnostic information in a way that results in non-optimal gambling-related judgments.

### Intuitive Numerators and Unused Ratios

Studies examining ratio bias may offer an explanation as to why people’s gambling-related judgments are influenced by a non-diagnostic piece of information when diagnostic information is present (Denes-Raj and Epstein 1994; Denes-Raj et al. 1995; Kirkpatrick and Epstein 1992). In the standard ratio bias paradigm, participants are tasked with selecting a “winning” red jelly bean from a bowl featuring both red and white beans. Importantly, participants are given two bowls to choose from: a large bowl that contains several red jelly beans (e.g., 7) with a small ratio of red to white beans (e.g., 7:93), and a small bowl that contains a single red jelly bean with a larger ratio of red to white beans (e.g., 1:9) and therefore a greater chance of making a winning selection. Surprisingly, participants often select from the large bowl, despite the fact that this bowl offers them a lower chance of making a winning selection (Denes-Raj and Epstein 1994). Even more surprisingly, some participants who self-report being aware that the small bowl is the optimal choice still prefer to choose from the large bowl. One explanation for this puzzling behaviour is that selecting from the bowl with the greatest number of winning jelly beans is of strong intuitive appeal, leading some participants to choose on the basis of a comparison between numerators (i.e., red jelly beans) instead of a comparison between ratios, resulting in a statistically suboptimal choice.

Applying this type of reasoning to a scratch card gambling domain, unclaimed prize information may similarly represent an intuitive piece of information (despite its non-diagnosticity) when compared to information involving the ratio of unclaimed prizes to total cards remaining (i.e., payback percentage). First, similar to the numerators in a ratio bias paradigm, unclaimed prize information focuses on the prizes that can be won, offering a seemingly attractive piece of information on which to base a decision. Second, using unclaimed prize information is an “easy” process involving only a simple comparison between two numbers (i.e., the number of prizes remaining), as opposed to a comparison between theoretical percentages (i.e., payback percentage). This may explain why people are so often drawn to using unclaimed prize information in their gambling-related decisions. Therefore, one may wonder whether increasing the intuitive appeal of payback percentage information (e.g., by making it easier to use) may increase the rate at which participants utilize this information and correspondingly optimize their decision making in a scratch card gambling scenario.

### Same Information, Better Decisions

There are many ways to represent numerical information. For example, in a gambling domain, one can state that a gamble has a 40% chance of success or that a gamble is successful four out of every ten times it is played. Normatively speaking, the way in which numerical information is presented should not influence the decisions made on the basis of this information. Nevertheless, research on the presentation of numerical information (primarily in the context of medical decision making and risk communication), has demonstrated that some formats may be more easily understood than others, leading to improvements in decision making. For example, the visual presentation of numerical information, such as in a graph or icon array, has been shown to improve judgments and decisions in various scenarios (Brase 2009; Galesic et al. 2009; Garcia-Retamero and Cokely 2013; Garcia-Retamero et al. 2010; Garcia-Retamero and Hoffrage 2013; Okan et al. 2012). This suggests that the understanding and proper use of numerical information can be improved simply by presenting it in a more intuitively appealing format.

### The Current Study

Past research has demonstrated that people remain unduly influenced by unclaimed prize information when making scratch card related judgments, despite being given additional relevant information (i.e., the total number of cards remaining) allowing them to discern each game’s true expected value (i.e., payback percentage). One possible reason for this behaviour is that participants may struggle to determine each game’s payback percentage by failing to properly combine both presented pieces of information. Therefore, eliminating the need for this calculation by explicitly presenting participants with the payback percentage value of each game may enable them to ignore non-diagnostic unclaimed prize information and instead base their judgments on meaningful payback percentage values. However, if unclaimed prize information is simply a more intuitively appealing type of information, due to it’s easy-to-use and prize-focused nature, then explicitly presenting the payback percentage of each scratch card game may not reduce the influence of unclaimed prize information. In Experiment 1, we investigated whether participants remained unduly influenced by unclaimed prize information when given the payback percentage of all scratch card games. In Experiment 2, we investigated whether graphically depicting payback percentage affects the use of this informative metric. Together, these studies will address how different information presentation formats may influence and possibly improve gambling-related judgments in the domain of scratch card gambling.

## Experiment 1

The primary goal of Experiment 1 was to examine whether participants would continue to be biased by unclaimed prize information when explicitly presented with a calculated payback percentage value for each scratch card. We predicted that participants would non-optimally align their scratch card preferences with intuitively appealing, yet non-diagnostic, unclaimed prize information, despite the inclusion of diagnostic payback percentage information. Specifically, we hypothesized that participants would feel more likely to win, be more excited to play, report a greater urge to gamble, and choose to hypothetically purchase a greater number of scratch cards with higher levels of unclaimed prizes—despite these cards having objectively lower expected values as depicted by payback percentage information.

## Method

### Participants

We pre-registered^1^ a sample size of 200 participants, a sample size that our previous research showed had sufficient power to detect biases due to unclaimed prize information. Ultimately, 201 participants were recruited from Amazon Mechanical Turk with the full sample collected prior to any data analysis. Participants were recruited under the condition that they were US residents and possessed a Mechanical Turk HIT approval rate greater than or equal to 95%. The present experiment took approximately 15 min to complete and participants were compensated $2.00 for their participation. All reported experiments received prior approval by the University of Waterloo Office of Research Ethics.

### Materials

#### Scratch Card Games

An image of a representative scratch card (100× Multiplier) was chosen from the Ontario Lottery and Gaming Corporation’s website (OLG 2018). Using Adobe Photoshop CS6, three versions of the same card were created (Green, Blue, and Red) by changing the colour of the scratch card. Specific information related to the number of top prizes and odds was removed from the card image as to not conflict with information that was presented within the experiment.

### Measures

#### Likelihood of Winning

Participants rated their likelihood of winning any prize while playing 100× Multiplier by responding to the following item: “How likely do you think you are to win a prize while playing 100× Multiplier?” Responses to this item were provided using a scale that ranged from 1 (*Extremely unlikely*) to 7 (*Extremely likely*).

#### Excitement

Participants stated their excitement to play each presented scratch card game by responding to the item: “How excited would you be to play 100× Multiplier?” Responses to this item were provided using a scale that ranged from 1 (*Not at all excited*) to 7 (*Extremely excited*).

#### Urge to Gamble

Participants reported their urge to gamble at various time points using the following item: “Please indicate your urge to gamble on 100× Multiplier.” Responses to this item were provided using a scale that ranged from 1 (*No urge to gamble*) to 7 (*Strong urge to gamble*).

#### Card Purchasing

We constructed a hypothetical card purchasing scenario in order to assess participants’ card purchasing behaviour as well as investigate any existing preferences between cards featuring varying levels of unclaimed prize and payback percentage information. Participants were asked to imagine that they had the opportunity to purchase any or all of the previously presented scratch card games at a price point of $5. Following these instructions, participants were asked how many of each card (100× Multiplier: Green, Blue, Red) they would like to purchase. For this item participants were allowed to hypothetically purchase anywhere from zero to seven cards for each scratch card game.

#### Problem Gambling Severity Index

The Problem Gambling Severity Index (PGSI; Ferris and Wynne 2001) is a subset of the Canadian Problem Gambling Index and provides a reliable and valid measure of problem gambling symptomotology. For this measure, participants completed 9 items addressing gambling-related harms on a scale from 0 (*Never*) to 3 (*Almost Always*). Each participants’ responses to individual items were summed to create an overall PGSI score. Scores of 0 on the PGSI indicate non-problem gambling, scores between 1 and 4 indicate low-risk gambling, scores between 5 and 7 indicate moderate-risk gambling, and scores of 8 and above are indicative of problem gambling (Currie et al. 2013). The PGSI was administered in order to characterize our sample. Thus, no specific predictions were made regarding the role of problem gambling symptomotology on unclaimed prize information bias.

#### Payback Percentage Usefulness and Understanding

Participants stated if they found prize payout information (i.e., payback percentage)^2^ useful and responded to two items assessing their understanding of this information. First, participants were asked “Did you find Prize Payout information useful when choosing between scratch cards?” and provided either a *Yes* or *No* response. Second, participants were provided with two statements describing payback percentage information, one assessing the overarching concept of payback percentage information and the other assessing participants’ understanding of how this information may change over the course of a scratch card’s lifetime. Participants responded to both of these items with either a *True*, *False*, or *Unsure* response. If participants responded with either a *True* or *False* response their confidence in their response was assessed on a 7-point Likert scale which ranged from 1 (*Not at all*) to 7 (*Extremely*). The exact wording of each of the three payback percentage related items can be found in the Supplementary Materials.

### Procedure

**Fig. 1:**
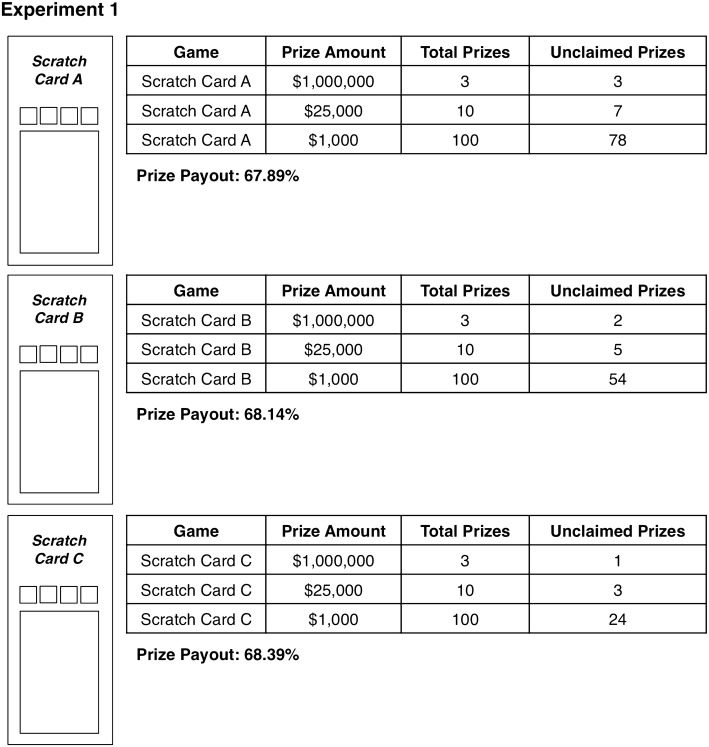
Schematic diagrams of the scratch card games and information tables presented to participants in Experiment 1. Note that “Prize Payout” refers to the payback percentage of the scratch card game

To begin the experiment, participants were introduced to three different versions (Green, Blue, and Red) of a scratch card game and given information that was common to all three game versions (e.g., top prize amount). These scratch card games were based on a scratch card available for sale in our home jurisdiction of Ontario (“100× Multiplier”; OLG 2018). Similarly, all accompanying information (e.g., payback percentage information) was representative of information that is presented with real-world scratch card games. After being introduced to all three versions of 100× Multiplier, participants were asked to provide baseline ratings for our likelihood of winning, excitement, and urge to gamble measures. Following these initial ratings, participants were provided with unclaimed prize and payback percentage information (labelled as prize payout information within our experiments) for each scratch card game and were given an explanation of each piece of information (see Fig. 1). Participants then provided a likelihood of winning, excitement, and urge to gamble judgment specific to each game. Next, participants completed our card purchasing measure where they indicated how many of each scratch card they would hypothetically elect to purchase (up to a limit of seven). Finally, to conclude the experiment, participants completed various demographic questions (i.e., age, gender, and scratch card gambling frequency), four Cognitive Reflection Test (CRT) items (taken from Primi et al. 2016; Toplak et al. 2014) the PGSI, and three items regarding payback percentage information. The CRT was administered for exploratory purposes and thus participants’ scores on this measure were not analyzed.

### Design

Participants were randomly assigned to one of nine conditions. Each condition differed in terms of which version of 100× Multiplier (Green, Blue, or Red) contained which amount of unclaimed prizes (low, medium, or high; our independent variable of interest). This counterbalancing ensured that any colour preferences that may be present in the sample were controlled for. Additionally, the order in which varying levels of unclaimed prize information was presented was counterbalanced, such that each amount of unclaimed prizes (low, medium, or high) was presented first, second, and third. Finally, payback percentage information was provided with each scratch card game and conflicted with unclaimed prize information, such that cards with a high level of unclaimed prizes featured a low payback percentage (i.e., 67.89%) and vice versa (see Fig. 1). Therefore, scratch cards with high levels of unclaimed prizes offered participants the lowest expected value, whereas scratch cards with low levels of unclaimed prizes offered the highest expected value (as made explicit by the payback percentage information presented).

### Data Analysis

Prior to analysis, data were cleaned such that all participants who failed to provide a response to one or more subjective judgment items were removed from the dataset. This resulted in eight participants being removed, leaving 193 participants in the final dataset. This data cleaning strategy was pre-registered on the Open Science Framework. The demographic characteristics of the sample can be viewed in Table 1. Participants’ likelihood of winning, excitement, and urge to gamble ratings across each level of unclaimed prize information were averaged and compared with a repeated-measures analysis of variance (ANOVA). In cases where the sphericity assumption was violated a Greenhouse–Geisser correction was applied to the degrees of freedom.

**Table 1:** Descriptive statistics

Measure	Experiment 1	Experiment 2
Age [mean (SD)]	35.58 (10.08)	35.33 (10.51)
Gender [*n* male, *n* female]	102, 90	119, 81
Frequency of scratch card gambling [*n* (%)]		
Had not played	48 (25.3%)	70 (35.2%)
1–5 times	80 (42.1%)	68 (34.3%)
6–10 times	28 (14.7%)	27 (13.6%)
11–15 times	21 (11.1%)	19 (9.5%)
16–24 times	5 (2.6%)	7 (3.5%)
24 or more	8 (4.2%)	8 (4.0%)
Problem Gambling Severity Index [*n* (%)]		
Non-problem gambling	103 (54.5%)	109 (55.9%)
Low-risk gambling	56 (29.6%)	51 (26.2%)
Moderate-risk gambling	10 (5.3%)	7 (3.6%)
Problem gambling	20 (10.6%)	28 (14.4%)

Descriptive statistics for all measures presented in Experiments 1 and 2. Categories for the Frequency of Scratch Card Gambling item represent participants’ self-reported scratch card gambling frequency in the last 12 months. Problem Gambling Severity Index category cut-offs are based on those provided by Currie et al. (2013)

## Results and Discussion

### Likelihood of Winning

**Fig. 2:**
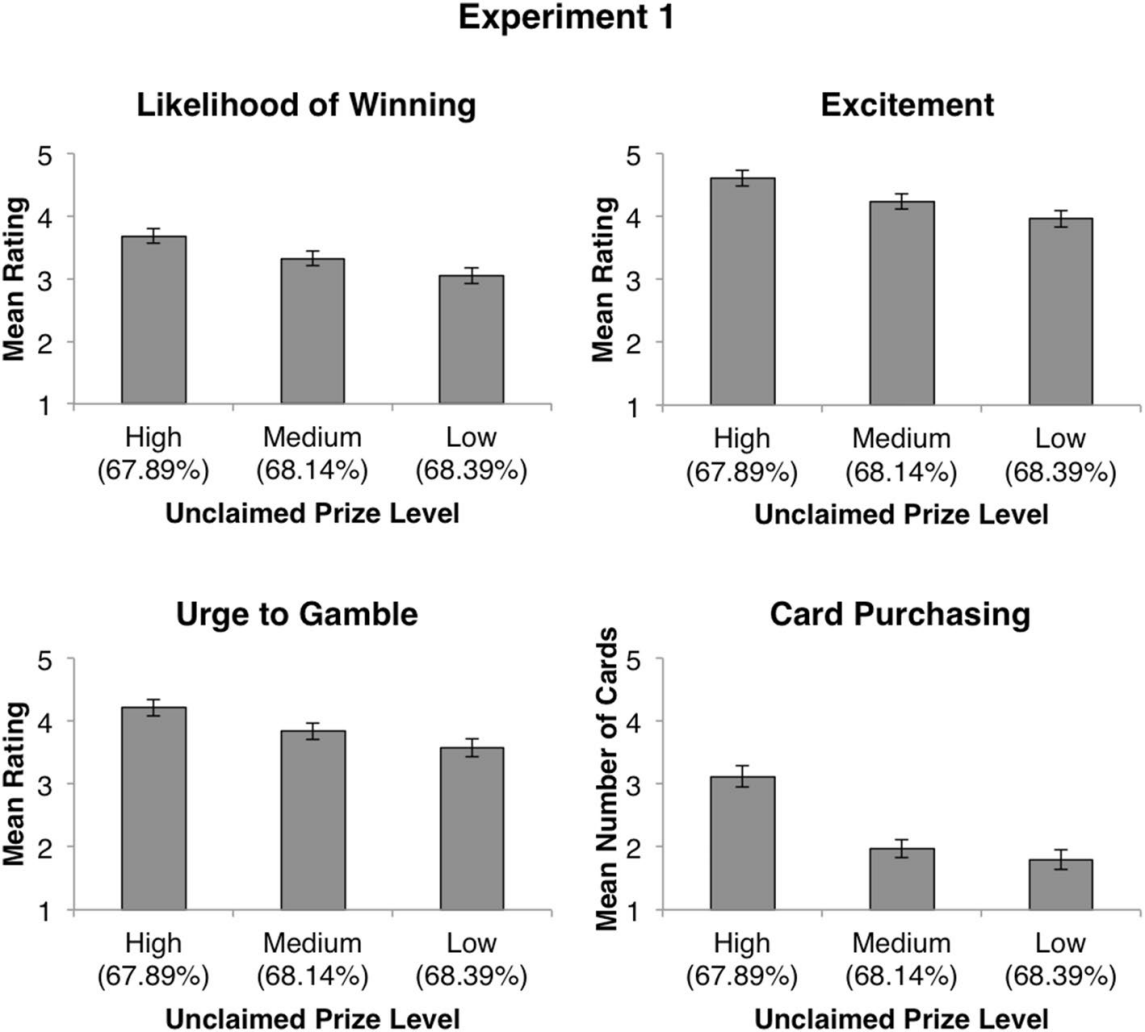
Results for Experiment 1. Mean values for ratings of likelihood of winning, excitement, urge to gamble, and number of cards hypothetically purchased at each unclaimed prize level (high, medium, and low). Payback percentage values presented at each level of unclaimed prize information are in parentheses. All error bars are ± 1 *SEM*

A repeated-measures ANOVA revealed a main effect of unclaimed prize information, *F*(1.73, 332.83) = 30.32, *p* < .001, $\eta_{p}^{2} = .14$ (see Fig. 2). Follow-up pairwise comparisons revealed significant differences between participant’s likelihood ratings for games with a high number of unclaimed prizes (*M* = 3.68, *SD* = 1.62) and a medium number of unclaimed prizes [*M* = 3.32, *SD* = 1.60; *t*(192) = 5.42, *p* < .001], significant differences between a medium number of unclaimed prizes and a low number of unclaimed prizes [*M* = 3.04, *SD* = 1.73; *t*(192) = 3.36, *p* = .001], and significant differences between a high number of unclaimed prizes and a low number of unclaimed prizes [*t*(192) = 6.70, *p* < .001]. Furthermore, participants’ baseline likelihood of winning ratings (*M* = 2.88, *SD* = 1.68) were shown to be significantly lower than their estimates for both medium and high levels of unclaimed prizes (*p* < .001 in both cases). No significant differences were found between participants’ baseline and low unclaimed prize estimates (*p* = .22).

### Excitement

A repeated-measures ANOVA revealed a main effect of unclaimed prize information, *F*(1.71, 327.41) = 27.28, *p* < .001, $\eta_{p}^{2} = .12$. Follow-up pairwise comparisons revealed significant differences between excitement ratings when there was a high number of unclaimed prizes (*M* = 4.60, *SD* = 1.73) and a medium number of unclaimed prizes [*M* = 4.24, *SD* = 1.72; *t*(192) = 5.03, *p* < .001], when there was a medium number of unclaimed prizes and a low number of unclaimed prizes [*M* = 3.96, *SD* = 1.81; *t*(192) = 3.34, *p* = .001], as well as when there was a high number of unclaimed prizes and a low number of unclaimed prizes [*t*(192) = 6.24, *p* < .001]. Furthermore, participants’ baseline excitement ratings (*M* = 4.51, *SD* = 1.72) were shown to be significantly higher than ratings for both medium [*t*(192) = 2.84, *p* = .005] and low [*t*(192) = 5.02, *p* < .001] levels of unclaimed prizes. No significant differences were found between participants’ baseline and high unclaimed prize ratings [*t*(192) = .98, *p* = .33].

### Urge to Gamble

A repeated-measures ANOVA revealed a main effect of unclaimed prize information, *F*(1.65, 317.65) = 29.54, *p* < .001, $\eta_{p}^{2} = .13$. Follow-up pairwise comparisons revealed significant differences between urge to gamble ratings given to scratch cards with a high (*M* = 4.21, *SD* = 1.79) compared to a medium (*M* = 3.84, *SD* = 1.85) amount of unclaimed prizes [*t*(192) = 4.99, *p* < .001], high compared to low (*M* = 3.57, *SD* = 1.93) amount of unclaimed prizes [*t*(192) = 6.34, *p* < .001], and medium compared to low amount of unclaimed prizes [*t*(192) = 3.71, *p* < .001]. Furthermore, participants’ urge to gamble on scratch cards with a high amount of unclaimed prizes was shown to be significantly greater than their baseline urge to gamble ratings [*M* = 3.68, *SD* = 1.84; *t*(192) = 6.21, *p* < .001]. No significant differences were found between participants’ baseline ratings and ratings given to medium or low unclaimed prize scratch cards (*p* = .058 and *p* = .211 respectively).

### Card Purchasing

Participants’ hypothetical card purchases across levels of unclaimed prize information were averaged and compared with a repeated-measures ANOVA. The overall ANOVA revealed a main effect of unclaimed prize information, *F*(1.62, 311.84) = 31.68, *p* < .001, $\eta_{p}^{2} = .14$. Follow-up pairwise comparisons revealed that participants more frequently chose to hypothetically purchase scratch cards with high numbers of unclaimed prizes (*M* = 3.12, *SD* = 2.38) compared to scratch cards with a medium [*M* = 1.97, *SD* = 2.02; *t*(192) = 6.50, *p* < .001] or low [*M* = 1.80, *SD* = 2.21; *t*(192) = 6.10, *p* < .001] number of unclaimed prizes. No significant difference was found when comparing hypothetical purchases for medium and low unclaimed prize scratch cards (*p* = .24).

### Payback Percentage Usefulness and Understanding

**Fig. 3:**
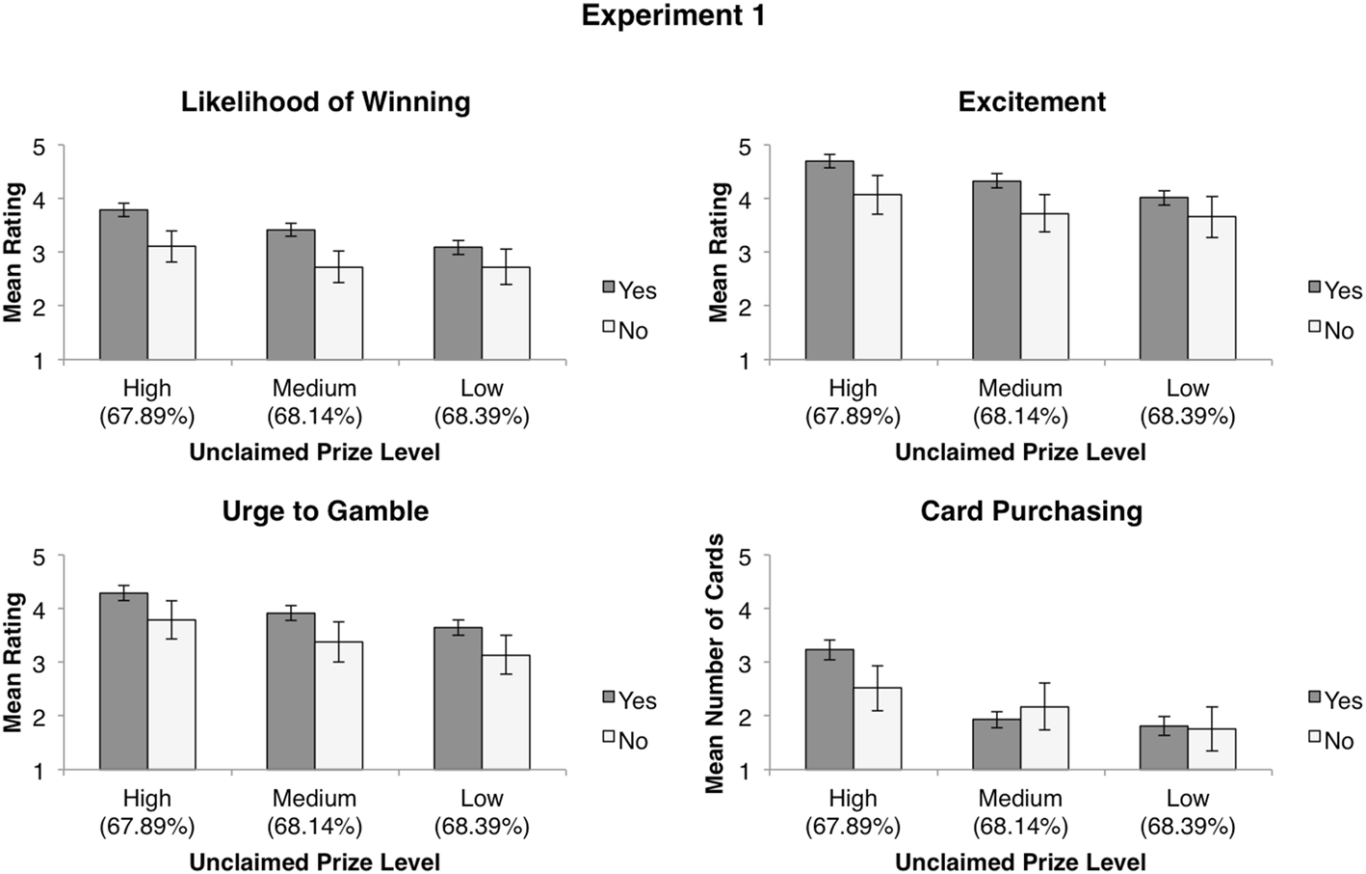
Mean values for ratings of likelihood of winning, excitement, urge to gamble, and number of cards hypothetically purchased at each unclaimed prize level split by whether or not participants indicated that they found payback percentage information useful when choosing between scratch cards. All error bars are ± 1 *SEM*

Participants’ responses to items assessing how useful they found and how well they understood payback percentage information (presented as prize payout information) are summarized in Table 2. Interestingly, the majority of participants (85.0%) stated that they found payback percentage information useful when choosing between scratch cards, despite their behaviour conflicting with this endorsement. To clarify the relationship between participants’ reports of payback percentage usefulness and their behaviour during the experimental task, we conducted an exploratory mixed factorial ANOVA with usefulness endorsement as the between-subjects factor and level of unclaimed prizes as the within-subjects factor. The results of this analysis revealed no significant interactions (*p* > .05 in all cases; see Fig. 3), indicating that participant’s endorsement of the usefulness of payback percentage information made no impact on their actual decision making behaviour in the experimental task. Correspondingly, an identical pattern of results was observed for both items assessing participants’ understanding of payback percentage information (all interactions *p* > .05).^3^ Therefore, regardless of participants’ endorsement or understanding of payback percentage information, unclaimed prize information biased responses such that participants displayed a preference for scratch cards with the lowest expected value (as represented by payback percentage) in our experimental task.

**Table 2:** Perceptions related to payback percentage information

Measure	Experiment 1	Experiment 2
Usefulness		
*n* Yes (%)	164 (85.0%)	161 (80.5%)
*n* No (%)	29 (15%)	39 (19.5%)
Definition		
*n* Correct (%)	122 (63.2%)	128 (64.0%)
*n* Incorrect (%)	19 (9.8%)	22 (11.0%)
*n* Unsure (%)	52 (26.9%)	50 (25.0%)
Change over time		
*n* Correct (%)	90 (46.6%)	106 (53.0%)
*n* Incorrect (%)	24 (12.4%)	36 (18.0%)
*n* Unsure (%)	79 (40.9%)	58 (29.0%)

Participants’ aggregate responses to items assessing the usefulness and understanding of payback percentage information in Experiments 1 and 2

## Experiment 2

In Experiment 2, we sought to investigate whether presenting payback percentage in an easy-to-use graphical format would affect the use of this informative metric. Specifically, we translated all numerical payback percentage values into a graphical star-based rating system, such that we depicted high payback percentages (i.e., 68.39%) with a greater number of stars (i.e., 5) and low payback percentages (i.e., 67.89%) with a lesser number of stars (i.e., 1). We believed that graphically depicting payback percentages in this way would make this information more intuitively appealing and easy-to-use, comparable to that of unclaimed prize information. Therefore, we predicted that this change in presentation format would increase the use of payback percentage information while simultaneously decreasing reliance on unclaimed prize information, resulting in more optimal choices in the form of increased preferences for higher value scratch cards. Specifically, we hypothesized that participants would no longer feel more likely to win, more excited to play, report a greater urge to gamble, or choose to hypothetically purchase a greater number of scratch cards with higher levels of unclaimed prizes.

## Method

### Participants

A sample of 201 participants was recruited from Amazon Mechanical Turk and received $2.00 upon completion of a 15 min online questionnaire. We pre-registered^4^ our intended sample size (*N* = 200) and collected our full sample prior to data analysis. All participants were recruited under the condition that they were US residents, had a 95% (or greater) HIT approval rate on Mechanical Turk, and had not taken part in Experiment 1.

### Materials

#### Scratch Card Games

Three scratch cards with different names and images were presented to participants in Experiment 2. These scratch cards were chosen from the Ontario Lottery and Gaming Corporation’s website (OLG 2018). We once again removed all information from each card image that conflicted with information that was presented within the experiment.

### Measures and Procedure

**Fig. 4:**
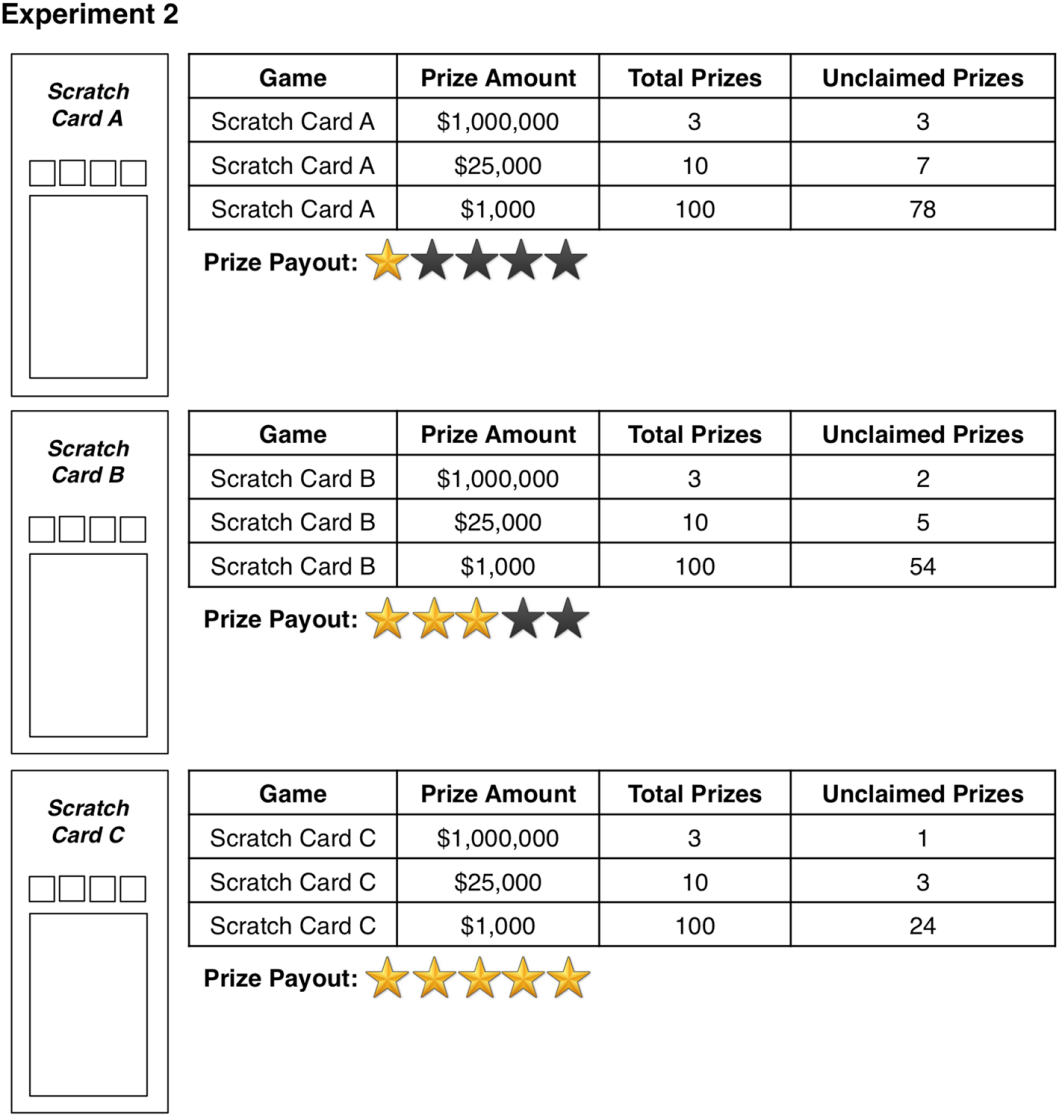
Schematic diagrams of the scratch card games and information tables presented to participants in Experiment 2. Note that “Prize Payout” refers to the payback percentage of the scratch card game (Color figure online)

In Experiment 2, payback percentage information was presented in a graphical format (see Fig. 4). That is, payback percentages of 67.89%, 68.14%, and 68.39% in Experiment 1 were represented with the graphical presentation of one, three, and five stars respectively in Experiment 2. Importantly, participants were instructed that the payback percentages for all presented games ranged from 67.89 to 68.39% ensuring that participants were prevented from interpreting differences in the graphical representation of payback percentage information as being larger compared to those in Experiment 1. With the exception of this change and the aforementioned change in the type of scratch card games presented, Experiment 2 featured the same measures and utilized an identical procedure as Experiment 1.

### Design

Participants were randomly assigned to one of nine conditions with each condition differing with regards to which of the three unique scratch card games contained which amount of unclaimed prizes (low, medium, or high). Thus, despite the change from Experiment 1 (using unique scratch card games as opposed to variations of the same game), this counterbalancing ensured that any scratch card preferences that may be present in the sample were controlled for. Additionally, as in Experiment 1, the order in which varying levels of unclaimed prize information was presented was counterbalanced, such that each unclaimed prize amount (and correspondingly, each magnitude of payback percentage information) was presented first, second, and third.

### Data Analysis

Prior to analysis, data were once again cleaned such that all participants who failed to provide a response to one or more subjective judgment items were removed from the dataset. This resulted in one participant being removed, leaving 200 participants in the final dataset. The demographic characteristics of the sample can be found in Table 1. As in Experiment 1, participants’ likelihood of winning, excitement, and urge to gamble ratings were averaged across varying levels of unclaimed prize information and compared with a repeated-measures ANOVA. In cases where the sphericity assumption was violated a Greenhouse–Geisser correction was applied to the degrees of freedom.

## Results and Discussion

### Likelihood of Winning

**Fig. 5:**
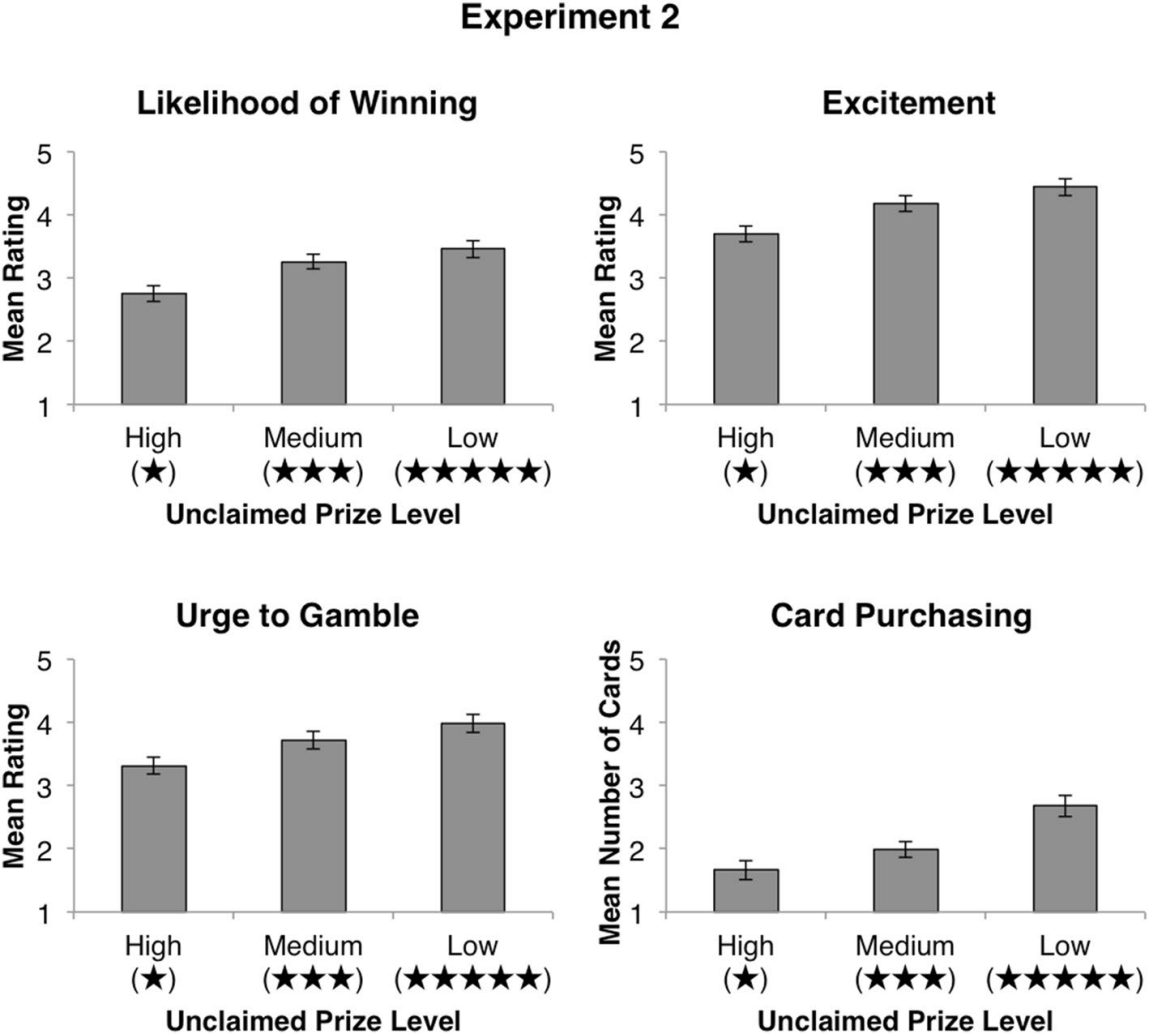
Results for Experiment 2. Mean values for ratings of likelihood of winning, excitement, urge to gamble, and number of cards hypothetically purchased at each unclaimed prize level (high, medium, and low). Payback percentage star ratings presented at each level of unclaimed prize information are in parentheses. All error bars are ± 1 *SEM*

A repeated-measures ANOVA revealed a main effect of unclaimed prize information, *F*(1.38, 275.04) = 17.73, *p* < .001, $\eta_{p}^{2} = .08$ (see Fig. 5). Unlike in Experiment 1, participants felt they were more likely to win playing scratch cards with fewer unclaimed prizes but greater payback percentages. Follow-up pairwise comparisons revealed significant differences between participant’s likelihood ratings for games with a low number of unclaimed prizes (*M* = 3.46, *SD* = 1.98) and a high number of unclaimed prizes [*M* = 2.75, *SD* = 1.76; *t*(199) = 4.47, *p* < .001], between a low number of unclaimed prizes and a medium number of unclaimed prizes [*M* = 3.26, *SD* = 1.65; *t*(199) = 2.05, *p* = .041], and between a medium number of unclaimed prizes and a high number of unclaimed prizes [*t*(199) = 4.94, *p* < .001]. Furthermore, participants’ baseline likelihood of winning ratings (*M* = 2.90, *SD* = 1.67) were shown to be significantly lower than ratings for both medium and low levels of unclaimed prizes (*p* < .001 in both cases). No significant differences were found between participants’ baseline and high unclaimed prize ratings (*p* = .22).

### Excitement

A repeated-measures ANOVA revealed a main effect of unclaimed prize information, *F*(1.56, 309.65) = 24.55, *p* < .001, $\eta_{p}^{2} = .11$, with participants reporting more excitement for scratch cards with fewer unclaimed prizes but greater payback percentages. Follow-up pairwise comparisons revealed significant differences between excitement ratings when there was a low (*M* = 4.44, *SD* = 1.90) and a medium number of unclaimed prizes [*M* = 4.18, *SD* = 1.76; *t*(199) = 3.01, *p* = .003], when there was a medium and a high number of unclaimed prizes [*M* = 3.70, *SD* = 1.85; *t*(199) = 4.93, *p* < .001], as well as when there was a low and a high number of unclaimed prizes [*t*(199) = 5.59, *p* < .001]. Furthermore, participants’ baseline excitement ratings (*M* = 4.60, *SD* = 1.73) were shown to be significantly higher than ratings for both high [*t*(199) = 8.85, *p* < .001] and medium [*t*(199) = 4.97, *p* < .001] levels of unclaimed prizes. No significant differences were found between participants’ baseline and low unclaimed prize ratings [*t*(199) = 1.68, *p* = .094].

### Urge to Gamble

A repeated-measures ANOVA revealed a main effect of unclaimed prize information, *F*(1.61, 319.95) = 22.60, *p* < .001, $\eta_{p}^{2} = .10$, with participants reporting a higher urge to gamble for scratch cards with fewer unclaimed prizes but greater payback percentages. Follow-up pairwise comparisons revealed significant differences between urge to gamble ratings given to scratch cards with a low (*M* = 3.98, *SD* = 2.08) compared to a medium (*M* = 3.72, *SD* = 1.98) amount of unclaimed prizes [*t*(199) = 3.13, *p* = .002], low compared to high (*M* = 3.31, *SD* = 1.93) amount of unclaimed prizes [*t*(199) = 5.47, *p* < .001], and medium compared to high amount of unclaimed prizes [*t*(199) = 4.46, *p* < .001]. Additionally, participants’ urge to gamble on scratch cards with a high and medium amount of unclaimed prizes was shown to be significantly lower than their baseline urge to gamble ratings (*M* = 3.88, *SD* = 1.96; *p* < .001 and *p* = .042 respectively). No significant difference was found between participants’ baseline ratings and ratings given to low unclaimed prize scratch cards (*p* = .25).

### Card Purchasing

Participants’ hypothetical card purchases across levels of unclaimed prize information were averaged and compared with a repeated-measures ANOVA. The overall ANOVA revealed a main effect of unclaimed prize information, *F*(1.49, 296.52) = 15.91, *p* < .001, $\eta_{p}^{2} = .07$. Follow-up pairwise comparisons revealed that participants more frequently chose to hypothetically purchase scratch cards with a low number of unclaimed prizes but higher payback percentage (*M* = 2.68, *SD* = 2.40) compared to scratch cards with a medium [*M* = 1.99, *SD* = 1.80; *t*(199) = 4.11, *p* < .001] or high [*M* = 1.66, *SD* = 2.10; *t*(199) = 4.41, *p* < .001] number of unclaimed prizes. Furthermore, participants preferred to hypothetically purchase scratch cards with a medium as opposed to a high number of unclaimed prizes [*t*(199) = 2.30, *p* = .022].

### Payback Percentage Information Usefulness and Understanding

**Fig. 6:**
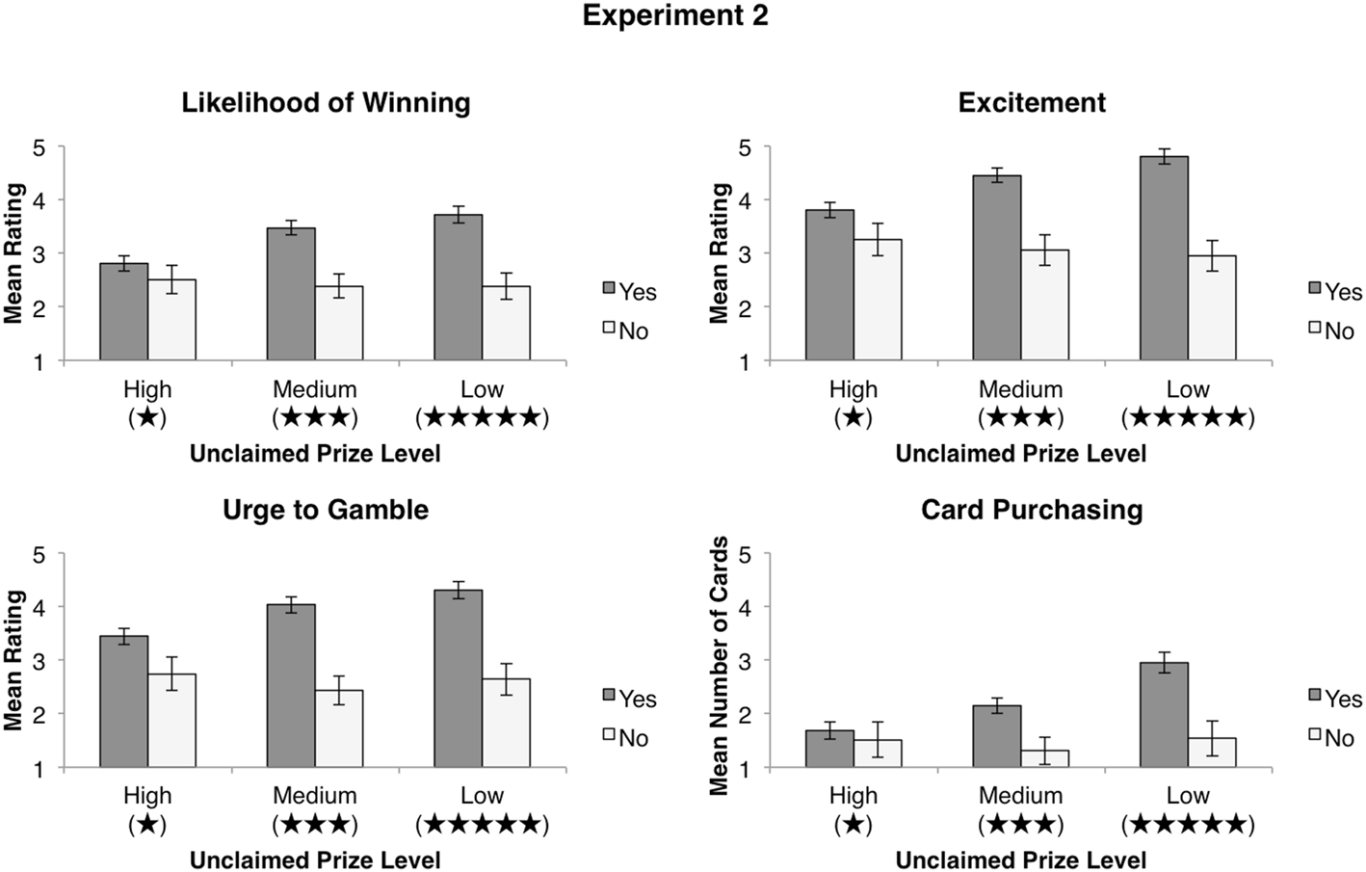
Mean values for ratings of likelihood of winning, excitement, urge to gamble, and number of cards hypothetically purchased at each unclaimed prize level split by whether or not participants indicated that they found payback percentage information useful when choosing between scratch cards. All error bars are ± 1 *SEM*

Participants’ responses to items assessing how useful they found and how well they understood payback percentage information (presented as prize payout information) are summarized in Table 2. As in Experiment 1, the majority of participants (80.5%) found payback percentage information useful when choosing between scratch cards. However, participants’ judgments were vastly different in Experiment 2, as they were no longer biased by unclaimed prize information in the presence of conflicting graphically depicted payback percentage information. As in Experiment 1, we performed an exploratory mixed factorial ANOVA with participant’s endorsement of payback percentage information usefulness as the between-subjects factor and the level of unclaimed prize information presented as the within-subjects factor. We observed an interaction between participants’ evaluations of the usefulness of payback percentage information and judgments made at each unclaimed prize level for all subjective categories and card purchasing (*p* < .05 in all cases; see Fig. 6). Follow-up analyses revealed significant main effects of unclaimed prize level for participants who endorsed payback percentage information as useful when choosing between scratch cards, but no such main effect for participants who did not find this information useful. Pairwise comparisons among the varying levels of unclaimed prizes for participants who endorsed payback percentage information as useful revealed significant differences between all levels of unclaimed prizes (*p* < .05 in all cases), such that these participants’ preferences were congruent with the value of all presented scratch cards.

Examining participants’ understanding of payback percentage information on judgments made at each unclaimed prize level revealed a significant interaction for all subjective categories (*p* < .05 in all cases).^5^ Follow-up analyses revealed significant main effects of unclaimed prize level for participants who correctly endorsed the definition of payback percentage as true, but no such main effect for participants who incorrectly endorsed this definition as false. Pairwise comparisons among the varying levels of unclaimed prizes for participants who correctly endorsed the definition revealed significant differences between all levels (*p* < .05 in all cases). The one exception to this overall pattern of results was participants’ card purchasing judgments, in which no interaction and only a marginal effect of unclaimed prize level was found (*p* = .059). Lastly, examining participants’ understanding of how payback percentage information can change over time on judgments made at each unclaimed prize level revealed no significant interactions for all subjective categories and card purchasing (*p* > .05 in all cases).

## General Discussion

The current set of experiments suggests that displaying payback percentage information in an easy-to-use graphical, as opposed to a numerical format, results in participants more optimally using the information presented to them. Specifically, when payback percentage information was displayed numerically, participants’ preferences were incongruent with the true expected value of the presented scratch cards, suggesting that they ignored the diagnostic payback percentage information and instead were influenced by the intuitive yet non-diagnostic unclaimed prize information. However, when identical payback percentage information was displayed in a graphical format, participants’ preferences were congruent with the true expected value of the presented scratch cards, suggesting that they utilized diagnostic payback percentage information and disregarded non-diagnostic unclaimed prize information. Interestingly, depicting payback percentage information in a graphical format did not increase participants’ endorsement of this information as useful, nor did it increase their understanding of it, despite the observed differences in behaviour. Therefore, the results of the current study demonstrate a way to improve the use of gambling-related information without increasing individuals’ knowledge of relevant concepts. Overall, this set of experiments suggests that participants’ judgments can be improved by presenting useful information in an easy-to-use and intuitively appealing format.

Previous research on unclaimed prize information bias (Walker et al. 2018) left open the possibility that this bias resulted from a calculation failure in which the majority of individuals simply did not have the ability to combine the presented pieces of information in a useful way (i.e., combining unclaimed prize information and ticket remaining information to calculate payback percentage). Therefore, providing individuals with the pre-calculated payback percentage of each scratch card game could have possibly reduced their dependence on non-diagnostic unclaimed prize information, by removing the barrier to acquiring payback percentage information. Furthermore, providing this calculated value simply reduces the effort needed to use payback percentage information and thus should theoretically have made this information more appealing to those utilizing a miserly cognitive strategy. Nevertheless, the results of Experiment 1 suggest that providing this pre-calculated value did nothing to reduce the influence of unclaimed prize information. Thus, when provided with a pre-calculated payback percentage, participants still do not properly utilize this information, leading to non-optimal scratch card preferences.

Although the majority of participants endorsed the usefulness of payback percentage information, their judgments were biased by the conflicting unclaimed prize information. Therefore, when these two pieces of information come into conflict, it appears that unclaimed prize information is favoured, despite being non-diagnostic, suggesting that it may be of greater intuitive appeal. One difference between these two pieces of information that may lead to unclaimed prize information being favoured is the fact that it is prize-focused. Research on ratio bias exhibits similar findings when conflicting the total number of winning items (e.g., 7 vs. 1) with the ratio between winning and losing items (e.g., 7:93 vs. 1:9; Denes-Raj and Epstein 1994). In this paradigm, people also non-optimally choose on the basis of prize-focused information as opposed to the ratio, thus leading to the speculation that prize-focused information is of high intuitive appeal and likely to influence judgment even when placed in conflict with more diagnostic information (Denes-Raj and Epstein 1994; Kirkpatrick and Epstein 1992).

In Experiment 2, we presented payback percentage in a graphical format, on the basis that graphical representations have been shown to improve decision making across a number of domains (Galesic et al. 2009; Garcia-Retamero and Cokely 2013; Garcia-Retamero et al. 2010). Consistent with this notion, we found participants’ judgments to be improved when payback percentage information was presented graphically, such that they more often displayed a preference for the scratch card with the highest value. Importantly, the graphic we utilized to convey payback percentage information was designed to be intuitive and easy-to-use.

These results suggest that our graphical presentation of payback percentage information (as opposed to its numerical counterpart) was in fact more intuitively appealing as participants’ preferences were consistent with this information over conflicting unclaimed prize information *only* when this information was presented in the graphical format. Furthermore, the use of stars to depict payback percentage information may have prompted a learned association with increasing numbers of stars being indicative of increasingly positive outcomes. If the presented graphical depictions were in fact more intuitively appealing compared to numerical formats and utilized previously learned associations, this may explain why participants utilized payback percentage information when presented graphically, but not numerically.

Another key difference between the presentation formats was the fact that the graphical depictions spanned the full scale of stars, whereas numerical information did not. That is, while cards with low levels of unclaimed prizes contained five times as many stars as cards with high levels of unclaimed prizes, the numerical percentage values used in Experiment 1 were not distributed as widely across the range of possible values. However, in Experiment 2 participants were explicitly instructed that all graphical depictions were restricted to a range of possible payback percentage values identical to those in Experiment 1. Despite this instruction, it is possible that the graphical presentation format served to exaggerate the differences in payback percentage values between cards, whereas the numerical presentation format resulted in relatively similar looking values. It remains unknown whether our manipulation would be as effective if both presentation formats were similarly distributed across a range of possible values (e.g., by depicting differences in payback percentage information using half-star differences instead of two-star differences).

The effectiveness of presenting payback percentage information graphically did not operate by increasing participants’ understanding of this information, nor did it increase how useful participants viewed this information. That is, across both experiments, participants endorsed the usefulness and understood the concept of payback percentage information to a similar degree. Nevertheless, despite endorsing this information as useful and correctly grasping the concept, participant’s preferences in Experiment 1 were incongruent with payback percentage information. Further, we found no interaction between participant’s responses to payback percentage information questions and their preferences between scratch cards. However, in Experiment 2, we did observe this interaction, such that participants who stated that they found payback percentage information useful and understood the concept were influenced by this information, and consequently displayed more optimal scratch card preferences. Instead of increasing the usefulness and understanding of payback percentage information, the benefits of using a graphical presentation may work by increasing the intuitive appeal of payback percentage information, such that when in conflict with contradicting unclaimed prize information, it becomes more influential for participants’ preferences.

Overall, it appears that understanding and recognizing the usefulness of payback percentage information is a necessary but not sufficient condition for optimal information use. This suggests that attempting to increase individuals’ understanding of payback percentage information, or other concepts like it, may be a surprisingly ineffective way to optimize decision making. Rather, this set of experiments demonstrates how modifying the gambling environment itself can lead people to better choices, akin to the use of nudges to improve outcomes in other contexts without directly increasing explicit knowledge (Thaler and Sunstein 2008). Thus, as this type of strategy appears demonstrably effective, it may offer a greater chance of success compared to traditional attempts which aim to increase individuals’ knowledge of important concepts such as payback percentage information.

In Experiment 2 participants received less precise information compared to participants in Experiment 1. Specifically, in Experiment 1, participants were given exact payback percentage information (e.g., 67.89%) for each scratch card where as in Experiment 2, participants were presented with a graphical display that corresponded to an ambiguous payback percentage value within a given range (i.e., 67.89–68.39%). Thus, an interesting observation in Experiment 2 was that participants produced more optimal preferences when given less precise information. This perplexing finding is not unprecedented in the judgment and decision making literature, as numerous studies have demonstrated that being provided with more detailed information does not necessarily improve decision making (Czerlinski et al. 1999; Dawes and Corrigan 1974; Gigerenzer and Brighton 2009; Gigerenzer and Goldstein 1996), reinforcing the idea that humans are not perfectly rational and unbiased decision makers.

### Implications

The results of Experiment 1 suggest that people may fail to understand and properly utilize complex statistical information presented within a gambling domain. However, as demonstrated in Experiment 2, presenting this information in an easy-to-understand graphical format can help improve peoples’ gambling-related judgments by helping to align their preferences with the true value of presented scratch cards. Therefore, one implication of the current study is that decisions made within the scratch card gambling domain may be improved if lottery operators providing prize payout information for these games also depict this information in an easy-to-understand graphical format. Nevertheless, the current study represents the first investigation into the effectiveness of using graphical depiction to represent complex statistical information in a way that improves gambling-related judgments. Therefore, future studies examining this approach in real-world gambling scenarios should be undertaken prior to incorporating this approach within a responsible gambling framework.

### Limitations

One limitation of the current investigation is the hypothetical nature of the scratch card gambling scenario that participants engaged in. Future studies should examine the effect of different presentation formats in real world gambling scenarios, allowing these findings to be generalized to a broader gambling context. Furthermore, the payback percentage values used in the current study constituted a fairly narrow range of possible values (i.e., only differed by .5%). However, this range of values is representative of actual payback percentage information for real scratch card games. Additionally, it is worth noting that payback percentage information is not always available for scratch games in a point of sale context (depending on the jurisdiction), but rather is available online from most lottery operators. Finally, the analyses examining the influence of payback percentage information usefulness and understanding on participants’ judgments were exploratory in nature, and therefore should be replicated.

### Future Directions

In the current study we demonstrate, within a scratch card gambling domain, how simply changing the format of presentation from complex numbers to easy-to-use graphics can improve peoples’ use of statistical information and optimize their scratch card preferences. Future studies should investigate how varying the way in which information is presented in other gambling domains may similarly improve gambling-related preferences. Furthermore, it is possible that certain gambles will become less appealing when information related to the gamble (e.g., payback percentage or odds of winning) is presented in a format that highlights the negative expected value present in the majority of gambles. Thus, varying information presentation formats should be explored as a possible responsible gambling tool to decrease harmful gambling behaviours. One potentially promising way of achieving this goal is through the use of icon arrays. Using this format for unclaimed prize information may be a way to present this information in a non-prize-focused way. That is, participants would simultaneously get a snapshot of all winning and losing cards, possibly providing an intuitive way to represent the ratio between winning and losing cards. Thus, icon arrays may provide a way of reducing the influence of non-diagnostic pieces of information and reducing harm in the domain of gambling.

## Conclusions

The current investigation demonstrates that displaying payback percentage information using an intuitively appealing graphic results in participants more optimally utilizing this information leading to improved scratch card preferences. Interestingly, graphically presented payback percentage information optimized participants’ scratch card preferences without increasing their understanding of payback percentage as a concept or their endorsement of how useful this information was. The results of the current study suggest that an understanding of payback percentage, or having this information presented graphically, are not sufficient in isolation to generate optimal scratch card preferences; however, when combined, preferences become congruent with this diagnostic piece of information. In conclusion, how information is presented has consequences: the results of our investigation point to certain graphical presentations as quick and easy ways to improve people’s use of gambling-related information, and subsequently their gambling-related judgments.

## Electronic supplementary material

Below is the link to the electronic supplementary material.

Supplementary material 1 (DOCX 14 kb)

## Footnotes

1 The pre-registration for Experiment 1 was completed through Open Science Framework and is accessible at the following link: https://osf.io/hmuzt/?view_only=18b772785d674d6f8e4a4d0c361f881e.

2 In both experiments, payback percentage information was referred to as “prize payout” information. This reflected the language used by the lottery operator in our home jurisdiction of Ontario, Canada. For clarity, we will refer to this information as payback percentage in the main body of the manuscript.

3 For these analyses, participants who responded as *Unsure* to the understanding questions were removed from the respective analysis, therefore leaving us only with participants who responded definitively (i.e., *True* or *False*).

4 The pre-registration for Experiment 2 was completed through Open Science Framework and is accessible at the following link: https://osf.io/bua4m/?view_only=1e07c044f51242ff8964447936a408b4.

5 As in Experiment 1, participants who responded as *Unsure* to the understanding questions were removed from the analysis.
