# Supplementary material for: Graphical Depiction of Statistical Information Improves Gambling-Related Judgments
Source: J Gambl Stud. 2019 May 27;35(3):945–68. doi: 10.1007/s10899-019-09860-1 (PMC6679834; doi:10.1007/s10899-019-09860-1)
Supplement: Supplementary file 1 — Supplementary material 1 (DOCX 14 kb) [file 10899_2019_9860_MOESM1_ESM.docx]

Supplementary Materials

For:

**Graphical Depiction of Statistical Information Improves Gambling-Related Judgments**

Alexander C. Walker^a^, Madison Stange^a^, Mike J. Dixon^a^, Derek J. Koehler^a^, and Jonathan A. Fugelsang^a^

^a^University of Waterloo

**Payback Percentage (Prize Payout) Usefulness and Understanding Items**

1. Did you find Prize Payout information useful when choosing between scratch cards? **(Yes; No)**

2. Prize Payout information represents the percentage of money spent that is paid out in prizing assuming that all scratch cards are purchased and all prizes are claimed. **(True; False; Unsure)**

3a. Prize Payout information, as presented in the current study, represented a theoretical payout percentage calculated at the time of a game's launch. Therefore, the current payout percentage may have been different from the stated payout percentage as for each version of 100X Multiplier prizes had been claimed and scratch cards had been sold (potentially changing the current payout percentage of these games). **(True; False; Unsure)**

3b. Prize Payout information, as presented in the current study, represented a theoretical payout percentage calculated at the time of a game's launch. Therefore, the current payout percentage may have been different from the stated payout percentage as for each game prizes had been claimed and scratch cards had been sold (potentially changing the current payout percentage of these games). **(True; False; Unsure)**

**The response options for each item are presented in bold*

***Due to changes in the scratch cards presented, the wording of our third item varied slightly between experiments. Item 3a presents the exact wording as it was displayed to participants in Experiment 1 whereas Item 3b presents the exact wording as it was displayed to participants in Experiment 2.*
